# Nurses' Experiences and Perceptions of two Early Warning Score systems to Identify Patient Deterioration—A Focus Group Study

**DOI:** 10.1002/nop2.821

**Published:** 2021-02-27

**Authors:** Caroline S. Langkjaer, Dorthe G. Bove, Pernille B. Nielsen, Kasper K. Iversen, Morten H. Bestle, Gitte Bunkenborg

**Affiliations:** ^1^ Department of Emergency Medicine Nordsjaellands Hospital University of Copenhagen Hilleroed Denmark; ^2^ Department of Cardiology Herlev and Gentofte Hospital University of Copenhagen Herlev Denmark; ^3^ Department of Emergency Medicine Herlev and Gentofte Hospital University of Copenhagen Herlev Denmark; ^4^ Department of Clinical Medicine University of Copenhagen Copenhagen Denmark; ^5^ Department of Anaesthesiology and Intensive care Nordsjaellands Hospital University of Copenhagen Hilleroed Denmark; ^6^ Department of Anesthesiology Holbaek Hospital Holbaek Denmark; ^7^ Department of Regional Health Research University of Southern Denmark Odense Denmark

**Keywords:** clinical assessment, clinical decision‐making, focus groups, Individual Early Warning Score, National Early Warning Score, nursing, patient deterioration, qualitative research, track and trigger systems, vital signs

## Abstract

**Aims:**

To explore Registered Nurses' experiences and perceptions with National Early Warning Score and Individual Early Warning Score to identify patient deterioration.

**Design:**

A qualitative exploratory design.

**Methods:**

Six focus groups were conducted at six Danish hospitals from February to June 2019. Registered Nurses from both medical, surgical and emergency departments participated. The focus groups were analysed using content analysis.

**Results:**

One theme and four categories were identified. Theme: Meaningful in identifying patient deterioration but causing frustration due to lack of flexibility. Categories: (a) Inter‐professional collaboration strengthened through the use of Early Warning Score systems, (b) Enhanced professional development and communication among nurses when using Early Warning Score systems, (c) Detecting patient deterioration by integrating nurses' clinical gaze with Early Warning Score systems and (d) Modification and fear of making mistakes when using Early Warning Score systems.

## 1 INTRODUCTION

Deterioration is a risk to all in‐hospital patients and includes the risk of suffering a serious adverse event (SAE) such as cardiac arrest, unplanned admission for intensive care, and unexpected death. The majority (84%) of patients have abnormal vital signs prior to SAE, suggesting that some can be prevented if abnormal vital signs are detected and acted upon by clinicians (Kause et al., 2004). Early Warning Score (EWS) systems comprise a tool for scoring vital signs and an escalation protocol. EWS systems have been implemented in healthcare systems worldwide to support the detection of abnormal vital signs and thereby help clinicians prevent patient deterioration and SAE (Jones et al., 2011). The intention is to standardize clinical monitoring and be an aid to clinical assessment and decision‐making (Royal College of Physicians, 2017).

The danger when using EWS systems is over‐relying on the system instead of using it as an aid or supplement to clinical assessment (Grant, 2019). Over‐reliance on the system has established a culture where monitoring vital signs are ritualistic and task‐oriented, and compliance with EWS systems is poor (Credland et al., 2018). Even though EWS systems are well established in many healthcare systems, continuous optimization and development of these systems are essential to support patient safety in healthcare systems that are constantly changing.

### 1.1 Background

Registered Nurses (RNs) play an essential role in recognizing and responding to patient deterioration because a major part of nursing practice is to observe, measure, document and react to signs of deterioration. However, recognizing and responding to patient deterioration is found to be complex, challenging and multifaceted (Credland et al., 2018). Using an EWS system is part of clinical practice in many healthcare systems including the Danish healthcare system. Since 2012, the British National Early Warning Score (NEWS) has been adopted to Danish critical care settings and is now used in all hospitals in the eastern part of Denmark. NEWS is a widely accepted system and has been found superior to other EWS systems in predicting SAE (Smith et al., 2013). A score is assigned to each of the following physiological vital signs; systolic blood pressure, heart rate, temperature, oxygen saturation, respiratory rate, level of consciousness and oxygen supplementation. The escalation protocol specifies the time interval for the next risk assessment and which actions to be taken by the nursing staff. Furthermore, the escalation protocol defines when to include the physician or activate the Medical Emergency Team or the Rapid Response Team (Royal College of Physicians, 2017).

National Early Warning Score is being referred to as a “one‐size‐fits‐all”‐system because it is used for all adult patients and does not consider the heterogeneity of patients or the causes of admission, medical history, chronic diseases, age and sex (Grant & Crimmons, 2018). In an attempt to meet these challenges, Individual Early Warning Score (I‐EWS) was developed in 2018. I‐EWS is based on the same vital signs and relates to the same escalation protocol as NEWS. However, the scores can be adjusted with a maximum of −4 or + 6 points by nursing staff based on their clinical assessment (Nielsen et al., 2020). I‐EWS has been compared to NEWS in a Danish cluster‐randomized, crossover study (estimated *N* = 150.000) with all‐cause mortality at 30 days as the primary endpoint (Nielsen et al., 2020). The focus group study presented in this paper relates to the cluster‐randomized study and contributes with knowledge and insight into RNs' experiences and perceptions with using both NEWS and I‐EWS to identify patient deterioration.

Introducing an EWS system is a complicated process, and it requires consideration of a range of factors interacting at different levels like individual healthcare professionals, organization, economic, social and political context (Connolly et al., 2017). When an EWS system was introduced into the Danish healthcare system, the primary focus was on standardizing monitoring practices and little focus was given to the intention to support RNs' clinical assessment and decision‐making (Bunkenborg et al., 2014). Considerations of how EWS systems can foster support from the end‐users, such as RNs, and ensure that their opinions are integrated are important. The absence of this may result in the potential benefits for patient safety and quality of care not being realized (Connolly et al., 2017). It can be argued that the introduction of an EWS system into the Danish healthcare system did not consider this complicated process. This may be one of the reasons why a culture has been established where monitoring vital signs is ritualistic and task‐oriented, and compliance with EWS systems is poor (Credland et al., 2018). Nevertheless, the importance of identification of patient deterioration will continue to increase in hospital wards as the population becomes older and sicker with more complex care needs. EWS systems need to be optimized and developed to support the complex care needs and patient safety, but there is a lack of knowledge about RNs' experiences and perceptions of these systems. This knowledge is important as the effectiveness of such systems is dependent on its users (Jensen et al., 2019). Therefore, this focus group study was designed to answer the following research question: how do RNs experience and perceive using NEWS and I‐EWS in their practice to identify patient deterioration?

## 2 AIM

This focus group study aimed to explore and understand RNs' experiences and perceptions of using NEWS and I‐EWS in their practice to identify patient deterioration.

## 3 DESIGN

A qualitative exploratory design using focus groups was applied. Focus groups can facilitate a semi‐structured discussion and group interaction that clarifies individual and shared perspectives about the topic.

## 4 METHODS

### 4.1 Sample and participants

Purposive sampling was used to recruit a total of 45 RNs from six Danish regional and university hospitals in the Capital Region of Denmark and Region Zealand. The recruitment was based on the five items and dimensions of information power (Malterud et al., 2016) and the following criteria: (a) RNs using NEWS or I‐EWS in their nursing practice, (b) RNs from both medical, surgical and emergency departments and (c) RNs who were willing to share their experiences and perceptions with using NEWS and I‐EWS. The RNs were recruited through local research staff. An over recruitment of 10% was done in case of cancellations. Three RNs were prevented from participating.

### 4.2 Data collection

**FIGURE 1:**
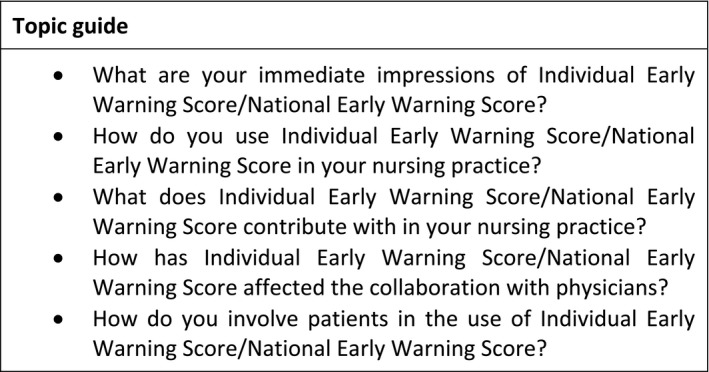
Examples of questions from the topic guide

Six focus groups were conducted from February to June 2019 at six hospitals and took place in undisturbed rooms located at the hospitals. A topic guide was followed starting with a broad question, before asking the focal questions (Figure 1). Three of the focus groups were conducted at hospitals using I‐EWS, two were conducted at hospitals using NEWS and the last one was conducted at a hospital where they had returned to NEWS after using I‐EWS. This was due to the crossover design of the aforementioned cluster‐randomized study (Nielsen et al., 2020). The first author (CL) facilitated the focus groups, and the second author (DB) and the last author (GB) participated as co‐moderators taking notes and asking supplementary questions. The focus groups were audio‐recorded and lasted between 50 and 62 min.

## 5 ANALYSIS

Content analysis was used to analyse the focus groups (Graneheim & Lundman, 2004). The focus groups were transcribed verbatim by the first author (CL) and organized with QSR NVivo 12^©^ software. CL and the last author (GB) divided the transcripts into meaning units, condensed meaning units and codes. The analysis began with a thorough reading of the transcripts and the identification of codes using an inductive approach. This was done individually with four of the six transcripts, and the remaining two transcripts were coded by CL. Finally, after coding all transcripts, CL and GB discussed until final coding was agreed upon. Sub‐categories and categories representing the manifest content of the focus groups were formulated independently. CL and GB discussed the categories until consensus was achieved. Furthermore, a theme representing the latent content of the focus groups was formulated in collaboration. The second author (DB) commented on the categories and the theme, which resulted in further abstraction.

## 6 ETHICS

### 6.1 Ethical considerations

The Helsinki Declaration was followed, and participants were included after oral and written informed consent had been obtained (World Medical Association, 1964). Participant anonymity and guaranteed confidentiality of any delivered information were emphasized as well as insurance about declining participation would not have managerial consequences. Information about the study, the authors and their interests in the topic had been sent out before study commencement. The study was presented to the Regional Ethics committee, and according to Danish law, no formal approval was needed (J. no. H‐18053090). The study data management has been approved by the Danish Data Protection Agency (J. no.: HGH‐2017–116, I‐suite no. 06,030).

### 6.2 Validity, reliability, and rigour

Lincoln and Guba's criteria for trustworthiness were used to assure rigour (Lincoln & Guba, 1985). Investigator triangulation contributed to credibility, confirmability and validation of the analysis. The authors have different credentials, some are experienced researchers within the field others are experienced qualitative researchers, ensuring that all the necessary competencies were present. The purposive sampling of participants made it possible to explore RNs’ individually and shared experiences (Graneheim & Lundman, 2004). Dependability and transferability were achieved by including information about the data collection and analysis as well as presenting quotations from the participants to illustrate the theme and categories.

## 7 RESULTS

### 7.1 Demographics characteristics

A total of 45 RNs participated. Forty participants were female, and 5 participants were male, the median age was 33 years with a range from 23–63, and the mean nursing experience was 9 years with a range from 0–31. Demographics characteristics from each of the focus groups are shown in Table 1.

**TABLE 1:** Demographics characteristics (*N* = 45)

Variable	Focus group 1	Focus group 2	Focus group 3	Focus group 4	Focus group 5	Focus group 6
Number of participants	9	8	6	7	7	8
Age in years						
Median	38	44.5	27.5	28	33	30.5
Range	30–55	25–54	24–63	25–46	23–59	24–55
Gender						
Female	6	8	5	7	7	7
Male	3	0	1	0	0	1
Experience in years						
Mean	10.7	11.6	3.6	6.2	12.7	7.3
Range	1–29	1–24	0–10	1–16	1–30	1–31
Departments						
Medical	3	1	3	6	6	3
Surgical	3	5	3	1	1	4
Emergency	3	2	0	0	0	1

### 7.2 Theme, categories and sub‐categories

**FIGURE 2:**
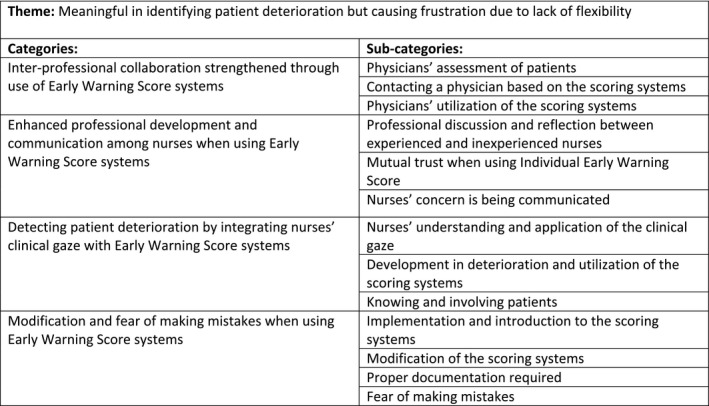
Theme, categories, and sub‐categories

One theme, four categories and thirteen sub‐categories were identified through the content analysis. The categories and sub‐categories were associated with the theme and with each other (Figure 2). NEWS and I‐EWS will be referred to as the scoring systems, unless it specifically concerns one of the two systems then NEWS or I‐EWS will be used in the following presentation of the findings.

#### 7.2.1 Meaningful in identifying patient deterioration but causing frustration due to lack of flexibility

The theme describes the latent content of the focus groups and during the analysis, a dualism appeared. RNs do consider scoring systems to be meaningful in identifying patient deterioration. The scoring systems facilitate the right help, and knowing the patients’ vital signs is an essential part of clinical assessments. However, an underlying issue also appeared about that the escalation protocol lacks flexibility. Especially when assessing patients with NEWS, RNs experienced that patients often needed to be observed more or less intensively than the escalation protocol dictated. As a result, RNs compensated by modifying and individualizing the scoring systems. I‐EWS did allow RNs to adjust the score, but they still had conflicting feelings and experienced that adjusting, modifying and individualizing the score was a deviation from the escalation protocol. The perception of deviating from the escalation protocol created frustration and a culture where RNs feared making mistakes even though they acted based on their observations and concerns of the patients. This restricted RNs' clinical practice and affected their use of the scoring systems.

#### 7.2.2 Inter‐professional collaboration strengthened through the use of Early Warning Score systems

There was an agreement among RNs that scoring systems improved the inter‐professional collaboration because their assessments and observations were an important part of the physicians’ assessment of patients. Furthermore, the scoring systems contributed to a shared language among RNs and physicians which made communication easier.

.. we have an argument that we can use, which is a shared language that our physicians use.. or understand and that we understand.. now the patient has a score of 8 based on this and this vital sign and two hours ago, the score was only.. or 6 hours ago the score was only 3 (RN from a surgical department with 31 years of experience)

Some RNs found it difficult to contact a physician solely based on a patient's score and dictated by the escalation protocol. These contacts were perceived as meaningless and a waste of time for both RNs and physicians. In contrast, when RNs had a concern about a patient and needed help or a treatment plan, they sometimes experienced that their concern was weighted less important than a high score by physicians. RNs simply missed a high score or deviating vital signs to support their concern.

You can call a physician and say I have this patient who is unstable in this and this way.. What is the score? But maybe the score is only 2 or.. but I see this and that and then it gets a little.. not neglected, that is a big word.. but because the score lacks to point out that there is something wrong, then it may not be as dangerous.. then it does not sound as dangerous as if the score was 6.. so the wording of it, is in some way missing.. (RN from a surgical department with 1 year of experience)

Physicians' use of the scoring systems had become an increasingly larger part of the daily work than earlier. RNs described how the scoring systems were used as a prioritization tool, so patients with the highest scores were seen first by the physicians.

So, you prioritize which patients should be seen first by the physician and in that way the scoring systems is clearly a helpful tool. (RN from an emergency department with 20 years of experience)

The inter‐professional collaboration was an important part of using the scoring systems according to RNs, and the scoring systems had strengthened both collaboration and communication. However, RNs still experienced challenges in inter‐professional collaboration.

#### 7.2.3 Enhanced professional development and communication among nurses when using Early Warning Score systems

Registered Nurses described how the scoring systems in general, but especially the scoring system incorporating RNs' clinical assessment (I‐EWS), supported and nudged professional discussions and reflections in the nursing group. Discussions of complex patient cases were highlighted by several RNs as a way to increase the quality of their clinical assessment of patients and subsequent care needs.

There was a lot more dialogue about these scores.. and nurses are also really reflecting a lot, I think.. their reflections are being articulated now where it previously would have been inside their heads.. you had a very good dialogue about what to do and how.. (RN from a medical department with 6 years of experience)

Registered Nurses agreed that scoring systems could be a helpful support for inexperienced RNs in identifying patient deterioration. However, professional discussions between RNs with different levels of experience were also important. More experienced RNs expected inexperienced RNs to ask them for advice if they were in doubt. It was emphasized that mutual trust was essential, especially when using I‐EWS, where RNs could adjust the patients’ scores. If RNs doubted each other's competencies, in terms of adjustment, it would create stress.

You need to trust each other as colleagues because if you are in doubt whether someone is adjusting some scores they should not adjust and you have to go and double‐check everything, then it will not work.. because it is stressful to go and check everything.. so it requires mutual trust among the staff.. (RN from an emergency department with 1 year of experience)

On the other hand, RNs experienced that adjusting the patients' score with I‐EWS was an easy and safe way to communicate important knowledge and their concern about patients' condition to their colleagues. This was not possible in the same way with NEWS.

I think the adjustments are a good way to communicate to your colleagues that I am more concerned about the patients than what the original score shows.. because often you are not able to do an oral handover in a busy everyday life. (RN from a surgical department with 3 years of experience)

The use of scoring systems contributed to professional development and communication. Mutual trust as well as professional discussions between inexperienced and experienced RNs was an important part of using both scoring systems, but especially when using I‐EWS.

#### 7.2.4 Detecting patient deterioration by integrating nurses' clinical gaze with Early Warning Score systems

When RNs discussed their experiences with identification of patient deterioration, clinical gaze was mentioned as an essential part of their clinical assessment of patients. Clinical gaze was defined as using one's senses, like touching the patient, feeling the pulse, looking after paleness and listening to the patient's breathing. Some RNs also described having a gut feeling that something was about to happen.

Just to touch and feel the patient on the forehead, feel an irregular pulse.. yes it also has something to do with the clinical gaze, look at the patient.. is the patient pale, blue, sweaty.. hear the breathing.. you use all your senses when you assess the patient.. (RN from a medical department with 5 years of experience)

Registered Nurses agreed that clinical gaze was an individual competence and that it could be used in different ways. The clinical gaze was also dependent on the ward and the patients, RNs worked with. The clinical gaze was described as a competence that built on experiences from past situations and required years of experience and training.

The clinical gaze needs to be trained.. you do not have it when you graduate from nursing school.. and it takes a certain number of years before you get it.. (RN from a medical department with 15 years of experience)

It became clear during the focus groups that there were various ways of using scoring systems. On some wards, only RNs did vital sign measurements, and on other wards, nursing assistants did it as well. Some wards used rounds where vital signs were measured at specific hours, even though the escalation protocol dictated otherwise, whereas other wards followed the escalation protocol punctually. Specific knowledge of the patient was also important when RNs aimed to identify patient deterioration. Without this knowledge, it could be difficult to adjust the score with I‐EWS. Involving patients in the scoring systems did not always happen systematically and was a balancing act. The vital signs could create a concern for patients if they did not have the right knowledge, but at the same time, patients could also help validate their vital signs if they knew what was habitual for them.

You do not go in‐depth with the vital signs with the patient.. it is just like that is fine, it is super nice or you have a low blood pressure, you have to drink some more water and that is just so.. there is someone, of course, it also depends on each patient how much they know.. some has been hospitalized for years, so they know how things should look.. it is really different, I think.. (RN from a medical department with 1 year of experience)

Registered Nurses stated that neither their clinical assessments, where the clinical gaze was an essential part nor scoring systems were sufficient in identifying deteriorating patients alone. It was the interaction of these two clinical tools that would support the identification. Utilization of scoring systems, the clinical gaze and involvement of patients differed from RN to RN and from ward to ward.

#### 7.2.5 Modification and fear of making mistakes when using Early Warning Score systems

There was no doubt that the purpose of introducing scoring systems was early detection and prevention of patient deterioration as well as improving patient safety. RNs expressed that it made sense and was not a waste of resources. The introduction of I‐EWS was a success at some wards, while others experienced some difficulties adapting a new scoring system and requested clear guidelines of how to use I‐EWS. In the daily work, NEWS could be rigid and was experienced as systematics only for the sake of systematics. RNs missed a greater integration of the clinical gaze in NEWS. Some of the more experienced RNs blamed the way that NEWS was implemented into the Danish healthcare system in 2012.

One of the problems with the way NEWS was implemented was that you did not pay much attention to the clinical gaze, but focused on systematic only.. then you sweep the other completely away, which I think is very problematic because it cannot stand alone, you need to use the scoring systems as a support to the clinical gaze and not the other way around. (RN from a surgical department with 24 years of experience)

Registered Nurses described how they found it necessary to modify and individualize the scoring systems when the escalation protocol and the defined cut‐off values did not match what they had observed assessing patients. With I‐EWS, RNs could adjust the score and integrate their clinical assessment, but with NEWS they described how they felt like deviating from the escalation protocol even though RNs measured the vital signs more often than required. Furthermore, the requirements for timely measurement and documentation could be difficult and frustrating to comply with. RNs understood why documentation was required but expressed that it could be challenging and wished that patients and relatives were a greater part of the documentation. One of the reasons why RNs thought that documentation was important was for their safety and to avoid any legal consequences. RNs described how the increasing focus on documentation had created a culture where RNs feared making mistakes and the subsequent consequences.

I think we are documenting to cover our backs.. if complaints should come further down the road, you have documentation that you have actually done something and have acted. (RN from a medical department with 3 years of experience)

Registered Nurses feared making mistakes and experienced that some of the scoring systems' requirements lacked meaning. However, most of the RNs were convinced that they possessed the right competencies and could identify patient deterioration.

## 8 DISCUSSION

Early Warning Score systems were in this study showed to be meaningful to RNs in identifying patient deterioration. This is in line with findings from other studies where RNs have adopted the scoring systems as useful tools to support patient safety and identify deteriorating patients (Jensen et al., 2019). However, the findings also illustrated that a lack of flexibility in the escalation protocol could cause frustration. The escalation protocol does not differentiate between types of disease or patients’ physiological response and NEWS has been referred to as a “one‐size‐fits‐all”‐system (Grant & Crimmons, 2018). The findings demonstrated that merely following protocols were not perceived as enough to ensure quality patient care. RNs felt responsible for more than just adhering to a system. This was in line with a study that found RNs using their professionalism, competences and clinical assessment along with objective vital sign measurements to build a clinical picture of the patient (Jensen et al., 2019). RNs experienced that I‐EWS, where their clinical assessment was integrated and the patient's score could be adjusted, was better than NEWS at supporting and nudging professional discussions and reflections in the nursing group. One explanation might be that RNs stated that using the clinical gaze was an essential part of their clinical assessment of patients, and neither their clinical assessments nor scoring systems were sufficient in identifying deteriorating patients alone. It was the interaction of these that would support the identification. RNs' clinical assessments, observations and concerns are an important part of identifying patient deterioration as it has been found to optimize care at an early stage of deterioration (Douw et al., 2016). This study also showed that EWS systems enhanced professional development and communication among RNs. Professional discussions between inexperienced and experienced RNs were an important part of using scoring systems and increased the quality of RNs' clinical assessments of patients and subsequent care needs. This is in line with several studies reporting that RNs collaborate with, support and help each other because they feel collectively responsible for patient care and for supporting their colleagues (Foley & Dowling, 2019; Jensen et al., 2019). It has also been suggested that EWS systems impact inter‐professional collaboration positively (Foley & Dowling, 2019). In this study, RNs experienced that the systems strengthened the inter‐professional collaboration and improved the communication between physicians and RNs because a shared language had been used. RNs did still experience some challenges in the inter‐professional collaboration when their concern was not weighted as important as a high score. Jensen et al., (2019, 2019) emphasize that physicians should listen to and respect RNs' clinical assessments of patients, which support our findings.

A perception or a misunderstanding was created among RNs because they felt like deviating from the escalation protocol when not followed punctually. This happened both when they measured patients’ vital signs more often than required or less than required. The escalation protocol recommends a minimum frequency of monitoring patients' vital signs, but the concern about a patient's clinical condition should always override this if it is considered necessary to escalate care (Royal College of Physicians, 2017). According to the intention with EWS systems, it is not a deviation from the escalation protocol when RNs measure patients’ vital signs more than required. It can be argued that it reflects good clinical practice as RNs use their clinical assessments and competencies to act on patients’ conditions. However, this perception or misunderstanding has created a culture where RNs feared making mistakes and where their clinical assessment was undermined. This occurred when the escalation protocol and the defined cut‐off values did not match what RNs had observed assessing patients. This restricted the RNs’ clinical practice and affected their use of the scoring systems. Previous studies have pointed out that compliance with NEWS and other EWS systems is poor (Credland et al., 2018), but without taking the RNs’ experiences and perceptions into account. Foley & Dowling (2019) found that RNs' clinical assessment can conflict with the escalation protocol and result in a task‐driven approach to the EWS systems instead of using them as an aid to clinical assessment and decision‐making. The perception or misunderstanding about EWS systems has not been highlighted before but gives insight and understanding of how RNs use EWS systems to identify patient deterioration. This needs to be integrated into future development and optimization of EWS systems if the compliance with EWS systems should increase as the effectiveness of such systems is dependent on its users (Jensen et al., 2019). Furthermore, the identification of patient deterioration requires ongoing training of RNs (Bunkenborg et al., 2013) and a behavioural change as well as a cultural shift to ensure patient safety (Foley & Dowling, 2019). The nursing profession, but especially the management implementing quality improvement systems and controls, needs to be aware that nursing practice with individual knowledge, experience and skills is being replaced by EWS systems (Grant, 2019). Using EWS systems as quality improvement systems are causing less attention on clinical assessments which can result in RNs over‐relying on the score instead of using their professional judgement and knowledge (McGaughey et al., 2017). Over‐reliance on these systems may impede RNs' ability to recognize patient deterioration because holistic patient assessments are being omitted and this often leads to poor compliance and delayed identification of patient deterioration (Credland et al., 2018). Furthermore, a quality improvement system with a high degree of standardization may contribute to an employee obligation rather than a professional responsibility, without improving the quality of care. The introduction of EWS systems may have been an oversimplified solution to the complex problem of preventing and detecting patient deterioration. Further research is needed to explore how the identification of patient deterioration which includes holistic patient assessments, professional reflections and discussions, and not only patients' vital signs, will affect the nursing practice and patient safety.

### 8.1 Limitations

Interviewer bias and the issue of giving the most socially acceptable response is potential study limitations when using focus groups. However, interviewer bias was controlled using co‐moderators, who compared to the facilitator were experienced researchers. Also, the issue of giving the most socially accepted response was met with making a safe atmosphere where everybody's experiences and perceptions were accepted. Member checking could potentially have enhanced the credibility of transcripts and results but would have been a lot of work for the participants and was not done because it was not considered essential to the validity of the study.

## 9 CONCLUSION

Findings demonstrate that EWS systems are meaningful to RNs in identifying patient deterioration, but the identification is complex. RNs' clinical gaze is an essential part of their clinical assessment of patients, and neither their clinical assessments nor scoring systems are sufficient in identifying deteriorating patients alone. It is the interaction of these that supports the identification. This is important to integrate into future development and optimization of EWS systems. Furthermore, the findings indicate that RNs' clinical assessments and observations must be weighted equally high as an EWS score because otherwise, it will contribute to a culture where clinical assessments and observations are neglected which can affect patient safety.

## CONFLICT OF INTEREST

No conflict of interest has been declared by the authors.

## AUTHOR CONTRIBUTIONS

CL, DB and GB: Conception and design, or acquisition of data, or analysis and interpretation of data. CL, DB, PN, KI, MB and GB: Drafting the manuscript or revising it critically for important intellectual content; final approval of the version to be published, participated sufficiently in the work to take public responsibility for appropriate portions of the content; accountable for all aspects of the work in ensuring that questions related to the accuracy or integrity of any part of the work are appropriately investigated and resolved.

## Data Availability

The data used to support the findings of this focus group study are available from the corresponding author on reasonable request.
